# Linc02349 promotes osteogenesis of human umbilical cord‐derived stem cells by acting as a competing endogenous RNA for miR‐25‐3p and miR‐33b‐5p

**DOI:** 10.1111/cpr.12814

**Published:** 2020-04-29

**Authors:** Lihua Cao, Wei Liu, Yancheng Zhong, Yanru Zhang, Dan Gao, Tiantian He, Ying Liu, Zi Zou, Yuqing Mo, Shuping Peng, Cijun Shuai

**Affiliations:** ^1^ NHC Key Laboratory of Carcinogenesis Hunan Cancer Hospital and The Affiliated Cancer Hospital of Xiangya School of Medicine Central South University Changsha China; ^2^ School of basic Medical Science Central South University Changsha China; ^3^ The Key Laboratory of Carcinogenesis and Cancer Invasion of the Chinese Ministry of Education Cancer Research Institute Central South University Changsha China; ^4^ Institute of Metabolism and Endocrinology Nation Clinical Research Center for Metabolic Diseases The Second XiangYa Hospital Central South University Changsha China; ^5^ Institute of Bioadditive Manufacturing Jiangxi University of Science and Technology Nanchang China; ^6^ State Key Laboratory of High Performance Complex Manufacturing Central South University Changsha China

**Keywords:** Linc02349, miR‐25‐3p and miR‐33b‐5p, osteogenic differentiation

## Abstract

**Objectives:**

Increasing evidences suggest that inducing mesenchymal stem cells to differentiate into osteoblasts has been as an especially important component in the prevention and therapy for degenerative bone disease. Here, we identify a novel lncRNA, linc02349, which increases significantly during osteogenic differentiation.

**Materials and methods:**

Human umbilical cord‐derived stem cells (hUC‐MSCs) and dental pulp mesenchymal stem cells were used. Overexpression and knockdown of linc02349 in cell lines were generated using lentiviral‐mediated gene delivery method. Bioinformatics prediction, Ago2‐RIP assay and dual‐luciferase reporter system were employed to examine miRNA which interacts with linc02349. The RNA FISH assay was performed to identify the subcelluar location of linc02349. Alizarin Red S staining, ALP staining and qPCR were applied to identify the osteogenic differentiation. The potential linc02349‐regulated genes, miR‐25‐3p and miR‐33b‐5p, were explored by ChIP, RIP and Western blotting assays. Micro‐CT was used to measure the osteogenic content in bone formation assay in vivo.

**Results:**

Linc02349 overexpression improves osteogenic differentiation by in vitro and in vivo analysis. Mechanistically, linc02349 acts as a molecular sponge for miR‐25‐3p and miR‐33b‐5p to control expression abundance of SMAD5 and Wnt10b, respectively, which eventually activated Dlx5/OSX pathway and hence promoted osteogenic differentiation. In addition, we revealed that STAT3 interacts with linc02349 promoter region and positively regulates the linc02349 transcriptional activity.

**Conclusion:**

These findings identify that linc02349 modulates the osteogenic differentiation through acting as a sponge RNA of miR‐25‐3p and miR‐33b‐5p and regulating SMAD5 and Wnt10b, and proposed a new interaction between STAT3 and linc02349, which could be a potential target in the process the osteogenesis of hUC‐MSCs for future clinical application.

## 1 INTRODUCTION

The regenerative medicine and tissue engineering are becoming the research hotspot in bone‐related disease such as osteoporosis. Osteoporosis is the result of the bone homeostasis tends to lose balance. Recent advances have demonstrated that inducing the bone formation to restore the normal bone homeostasis is efficient for osteoporosis treatment.^1^ Although osteogenesis induced by biological materials has gained much attention, classic methods are still applied in the studies of osteogenic differentiation of MSCs.^2^, ^3^, ^4^, ^5^ Human umbilical cord‐derived mesenchymal stem cells (hUC‐MSCs), a stem cell population, possess many advantages relative to other cell sources,^6^ including easily isolated, abundantly supply,^7^ low immune rejection,^8^ self‐renewing ability and multipotency.^9^ In previous studies, hUC‐MSCs displayed great potential for bone formation both in vitro and in vivo.^1^, ^10^, ^11^ Therefore, further work for exploring the molecular mechanism of the osteogenesis of mesenchymal stem cells becomes very important in the etiology and clinical treatment of bone‐related diseases.

Long noncoding RNAs (lncRNAs) are a class of transcripts longer than 200 nt,^12^ which have been shown to participate in cell cycle, pluripotency and reprogramming, etc^13^, ^14^, ^15^, ^16^ Notably, some lncRNAs are important contributors to regulate the osteogenic differentiation of MSCs and the efficacy of bone tissue engineering for bone repair.^17^ Thus, a more comprehensive understanding of the biology of lncRNA possibly reveals mechanisms of regenerative medicine for repairing bone tissue. However, only few lncRNAs have been characterized in osteogenesis, and the knowledge related to mechanism is also limited. Recent researches have shown that certain long noncoding RNA can act as ceRNA to regulate osteogenic differentiation of MSCs.^18^, ^19^ For instance, LINC00707 has been shown to stimulate osteogenesis through promoting Wnt2B expression by functioning as ceRNA for microRNA‐370‐3p.^20^ LncRNA KCNQ1OT1 contains functional miR‐214 target sites and can promote BMP2 expression to control osteogenic differentiation.^21^

In our previous study, we identified lncRNA profile during the osteogenic differentiation induction of hUC‐MSCs using Agilent Human lncRNA Microarray. A total of 23 lncRNA were significantly differentially expressed upon osteogenic differentiation.^22^ Among those, LOC100506530 (the alternative name of linc02349) was significantly increased. In this study, we revealed and demonstrated that linc02349 promotes osteogenesis of hUC‐MSCs by upregulating SMAD5 and Wnt10b via competitively sponging miR‐25‐3p and miR‐33b‐5p, which eventually activated Dlx5/OSX pathway. In addition, we investigated that STAT3 could bind to linc02349 promoter region and upregulate the linc02349 expression level. Taken together, these results reveal that linc02349 might be a potential target in the process of hUC‐MSCs osteogenic differentiation for future clinical application.

## 2 MATERIALS AND METHODS

### 2.1 Cell culture and differentiation

HUC‐MSCs (QC1205) and dental pulp mesenchymal stem cells were purchased from National Engineering Research Center for Human Stem Cells and cultured in normal culture medium, composing of DMEM/F12 and 10% FBS (Gibco). To initiate osteogenesis, hUC‐MSCs were seeded in 35 mm dish. Switch the medium to osteogenic‐induced medium, which consists of DMEM/F12, 10% FBS, 10^−7^ M dexamethasone, 10 mg/L ascorbic acid and 10 mmol/L β‐Glycerophosphoric acid disodium salt (Sigma) when cell density over 80%. The HEK 293 cells and HEK293T cells were cultured in DMEM medium added with 10% FBS.

### 2.2 Plasmid construction

Full length of linc02349 was subcloned into pcDNA3.1 (+) vector by Sangon Biontech Company. Human Wnt10b 3’UTR sequence, SMAD5 3’UTR sequence or linc02349 promoter sequence was subcloned into the pMIR‐REPORT^™^ vector. All of the cDNA sequences were from database searching (Ensemble and NCBI websites). The primers are listed in Table S1.

### 2.3 Stable cell lines construction

Linc2349 stable expressing hUC‐MSCs were generated using lentiviral‐mediated gene delivery method. To generate lentivirus, pCDH‐linc02349 plasmid or control plasmid was co‐transfected with two packaging vector PMD2.G and pspAX2 into HEK293T cells using Lipofectamine 2000 Reagent (Invitrogen). Lentivirus gene transfer system was also applied to produce shlinc02349 lentivirus. The supernatant medium was harvested and filtered by 0.45‐mm pore‐size filter (Sangon). All lentiviruses were used for hUC‐MSCs infection by adding polybrene (5 μg/μL) to the lentivirus. In order to measure infection efficiency, an inverted fluorescence microscope (Nikon) was used to calculate the percentage of GFP + cells.

### 2.4 Cell transfection

Cells were transiently transfected with (a) plasmid for linc02349, (b) validated si‐STAT3 (GenePharma), (c) miR‐25‐3p/33b‐5p mimics/inhibitors (GenePharma), (d) the lentiviral particles of pLVTH‐shlinc02349 or pCDH‐linc02349, and (e) corresponding negative control. The transfections were used Lipofectamine 2000 Reagent (Invitrogen) according to the manufacturer's instructions. The related siRNAs and shRNAs sequences were included in Table S1.

### 2.5 qPCR analysis

Total RNA from QC1205, HEK293 cells was lysed in TRIzol (QIAGEN) according to the user guide. 1.5 μg of purified RNA was reverse‐transcribed using the RevertAid First Strand cDNA Synthesis Kit (Thermo). The relative abundance of the mRNAs was quantified using 2 × SYBR Green qPCR Master Mix (Biotool) according to manufacturer's instructions. Primer sequences are listed in Table S1. All samples were performed in triplicate independent tests.

### 2.6 Western blot analysis

Western blot analysis was conducted as described previously.^23^, ^24^ In brief, total protein were harvested from cells with RIPA buffer (Beyotime) supplemented with cocktail (Beyotime). Then, the soluble protein was collected. A total of 50 μg of proteins was subjected to 10% SDS‐PAGE gel electrophoresis and transferred to a PVDF membrane (Millipore). The membranes were then blocked with 5% skimmed milk and probed with antibodies: GAPDH (Sangon Biotech), OPN (Cell Signaling Technology), OSX (Abcam), Runx2 (Cell Signaling Technology), SMAD5 (Cell Signaling Technology), Wnt10b (Abcam), Dlx5 (Cell Signaling Technology) and STAT3 (Abcam). The signal was visualized on MiniChemi 610 System Instrument (Sage Creation Science Co.). All samples were detected in triplicate independent tests.

### 2.7 Luciferase assay

HEK293T cells were seeded on a 24‐well plate, and the cells were allowed to grow until 80% cell density. Cells were then co‐transfected with pMIR‐linc02349 and miRNA mimics or inhibitors. To detect linc02349 promoter activity, siSTAT3 or si‐NC was co‐transfected with pMIR‐promoter‐linc02349 into HEK293T cells. To detect miRNA bind to 3’UTR of messenger RNA (mRNA) targets, pMIR‐SMAD5 3’UTR or pMIR‐Wnt10b 3’UTR was co‐transfected with miRNA mimics or inhibitors into cells, respectively. pRL‐TK (Renilla) vector as an internal control for normalization was co‐transfected in these experiments. Luciferase assay was measured by dual‐luciferase assays reagents (Promega) according to the manufacturer's protocol. All samples were detected in triplicate independent tests.

### 2.8 RNA immunoprecipitation (RIP)

1 × 10^7^ cells were lysed in RIP lysis buffer containing protease inhibitors and RNase inhibitor. Protein‐G/A plus agarose was incubated with antibody against Ago2 (Abcam) or NC rabbit IgG (Abcam) at 4°C for overnight. The precipitated RNAs was lysed with TRIzol Reagent, reverse‐transcribed using the RevertAid Kit (Thermo) and subsequently examined by qPCR. The primers used are listed in Table S1. All samples were detected in triplicate independent tests.

### 2.9 ALP activity and alizarin red staining

HUC‐MSCs were seeded in 35 mm dish and change the medium with osteogenic‐induced medium when cells density reaches 90%. The ALP staining was conducted on day 14 using ALP Kit (Beyotime) according to the manufacturer's instructions. The alizarin red staining was measured on day 28, and the hUC‐MSCs were washed with PBS and fixed with 4% paraformaldehyde for 0.5 hour, then stained with alizarin red staining solution (Sigma) according to the manufacturer’ s instructions. All samples were measured in triplicate independent tests.

### 2.10 RNA fish

The RNA fluorescence in situ hybridization (FISH) assay was performed following the protocol from BOSTER. To detect linc02349 RNA, Digoxin tag probe sequences against linc02349 and all related reagents were purchased from Sangon Biotech. UltraVIEW VoX (PerkinElmer) confocal fluorescence microscope was used to visualize samples.

### 2.11 Bone formation assay in vivo

This study was approved by the Animal Ethical and Welfare Committee of Central South University. The hUC‐MSCs transfected with pCDH‐linc02349 or pCDH‐control vector were induced in osteogenic medium at 37°C for 7 days. Then, the cells were harvested, and 2 × 10^6^ cells were incubated with Bio‐Oss Collagen scaffolds (5 × 5 × 1.75 mm^3^) (Geistlich) scaffolds for 1 hour, which were transplanted subcutaneously in right back of BALB/c nude mice(5 weeks old, male, n = 5 per group). After 8 weeks, the Bio‐Oss Collagen were obtained and fixed in 4% paraformaldehyde. The implants were analysed via Quantum GX micro‐CT Imaging System (PerkinElmer), with the Quantum Supporting Xcapture Software (PerkinElmer) used to analyse the BV/TV. After that, implants were decalcified in 10% EDTA for 14 days, embedded with paraffin and sliced into sections (4 μm thickness). The sections were stained with H&E (Boster) and Masson Trichrome (Solarbio). In addition, IHC staining was performed with anti‐OPN. The images were taken using CKX41 optical microscope (Olympus).

### 2.12 Chromatin immunoprecipitation (ChIP)

The hUC‐MSCs were culture in osteogenic‐induced medium, after 7 days cross‐linked with 1% formaldehyde at 37°C for 10 minutes, terminated with 0.125 mol/L Glycine (Bioshrap) for 10 minutes. Then, 2 × 10^7^cells were harvested and lysed in SDS lysis buffer supplemented with cocktail. The cell lysis was sonicated with Cole‐Parmer Instruments CP130 (Vernon) to shear chromatin, then centrifuged at 13 000 *g* for 15 minutes. The supernatants fixed with antiSTAT3‐antibody (Cell Signaling Technology), c‐JUN (Cell Signaling Technology), and normal rabbit IgG (Abcam), adding Protein‐A/G Immunoprecipitation Magnetic Beads (Biomake) for overnight at 4.0°C. After interacting 10% Chelex‐100 mixtures (Bio‐Rad) to reverse crosslinking, DNA was purified and examined by qPCR. The ChIP‐qPCR primer sequences are included in Table S1, and all samples were performed in triplicate independent tests.

### 2.13 Statistical analysis

Experimental data were analysed by graphpad prism 7 and presented as mean ± SD. Two groups of data were statistically analysed using Student's *t* test or one‐way ANOVA test. The results were considered to be statistically significant when *P*‐value < .05.

## 3 RESULTS

### 3.1 Linc02349 is upregulated during the osteogenesis of hUC‐MSCs

**Figure 1:**
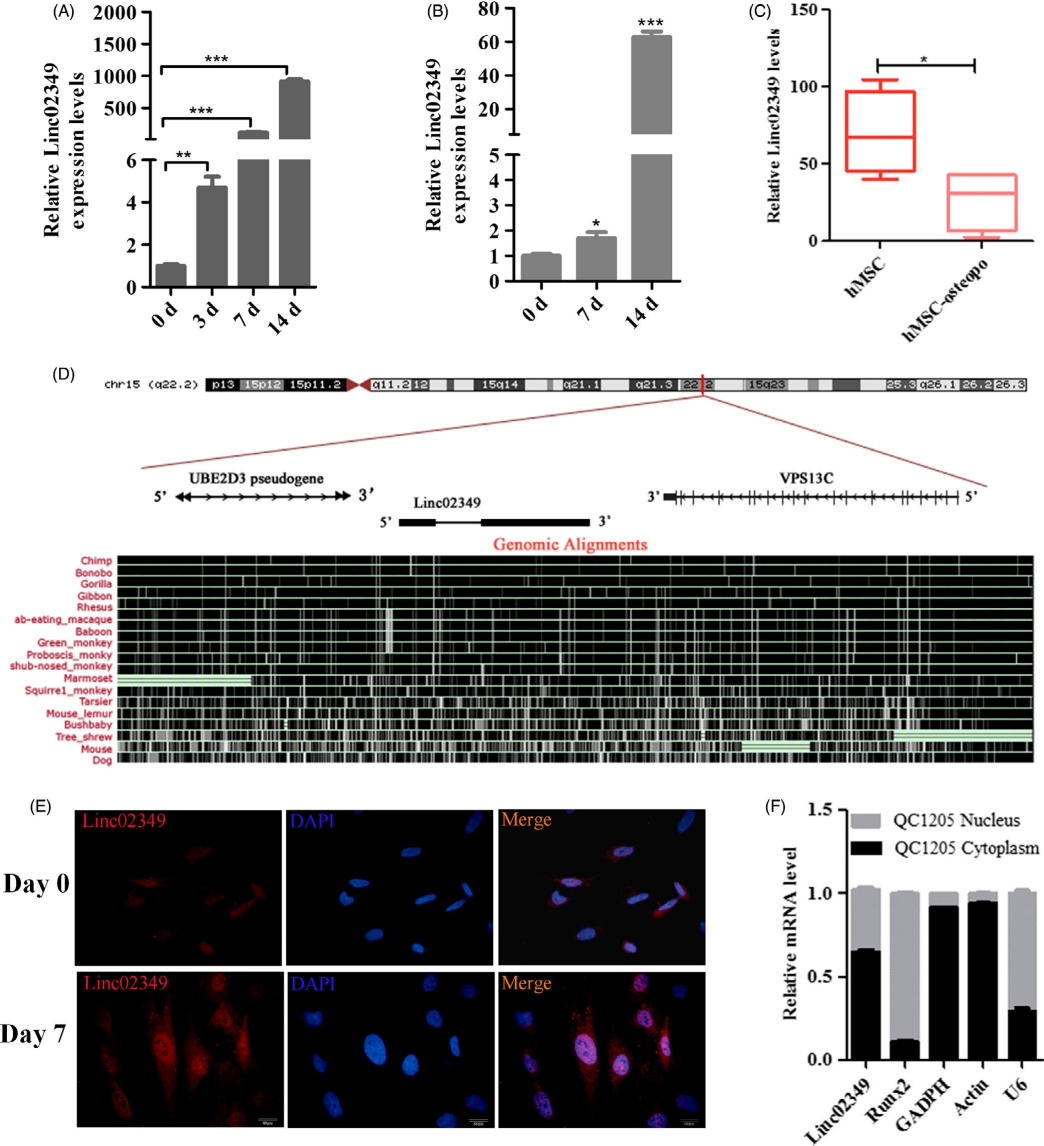
Linc02349 expression is upregulated during human umbilical cord‐derived mesenchymal stem cells (hUC‐MSCs) osteogenesis. A, qPCR showing the increasing expression of linc02349 compared with the hUC‐MSCs (day 0 cells) during the osteogenic differentiation. The data are shown as mean ± SEM, n = 3. **P < .01; ***P < .001. B, The expression level of linc02349 was upregulated compared with the dental pulp mesenchymal stem cells (day 0 cells) during the osteogenic differentiation. The data are shown as mean ± SEM, n = 3. *P < .05; ***P < .001. C, The mesenchymal stromal cells from non‐osteoporotic donors (hMSC group, 4 samples) revealed higher expression of linc02349 compared to patients suffering from osteoporosis ((hMSC‐osteopo group, 4 samples). The data were from GSE35958. Values are mean ± SD. *P < .05. D, The location (upper panel) and sequence conservation (lower panel) of linc02349 on the genome from UCSC website. It showed that linc02349 gene is located at 15q22.2 locus, between the UBE2D3 pseudogene and VPS13C genes, and it identified as a modestly conserved locus in primate species. RNA FISH assay (E) and nucleus/cytosol fractionation analysis with qPCR (F) showing linc02349 expressed in cytoplasm and nucleus. Blue, DAPI. Red, linc02349. Scale bar, 20μm. GAPDH and Actin served as positive reference for cytoplasmic gene expression. U6 and Runx2 served as positive reference for nuclear gene expression

In our previous study, to identify functional lncRNAs correlating with osteogenesis, lncRNA microarrays were applied to analyse lncRNA expression profiles.^22^ We revealed that 20 lncRNAs were upregulated and 9 were downregulated. Among these upregulated genes, we identified an uncharacterized, the most highly expressed lncRNA, termed LOC100506350 (as known as linc02349). To confirm whether linc02349 expressed consistently with the microarray data, we examined linc02349 expression pattern during osteogenesis. It is found that the linc02349 was increased gradually during hUC‐MSCs and dental pulp mesenchymal stem cells (Figure 1A,B). Meanwhile, the data from GSE35958 shown that lower expression level of linc02349 in mesenchymal stromal cells (hMSC) from patients suffering from osteoporosis compared with hMSC from non‐osteoporotic donors (Figure 1C).

The linc02349 gene is located at chromosomal locus 15q22.2 locus, between the UBE2D3 pseudogene and VPS13C genes, composed of two exons and spanned 963 nt lncRNA, and it identified as a modestly conserved locus in primate species (Figure 1D). We further confirmed that linc02349 was highly expressed in bone marrow, and expressed in MSCs and adipose tissues in a quite low level (Figure S1). We nextly examined its coding potentiality. We constructed a plasmid expressing full‐length linc02349 and HA tag.QPCR confirmed that the linc02349 and M3 (a positive control) expression level was higher in the HA‐linc02349 and HA‐M3 groups, while Western blotting revealed that no additional polypeptides were detected in HA‐linc02349 group suggesting it has no coding potentiality (Figure S2A‐C). Furthermore, cellular fractionation assays with qPCR analysis and RNA FISH assay showed that linc02349 is localized in the cell nucleus and cytoplasm (Figure 1E,F).

### 3.2 Linc02349 promotes osteogenic differentiation of hUC‐MSCs

**Figure 2:**
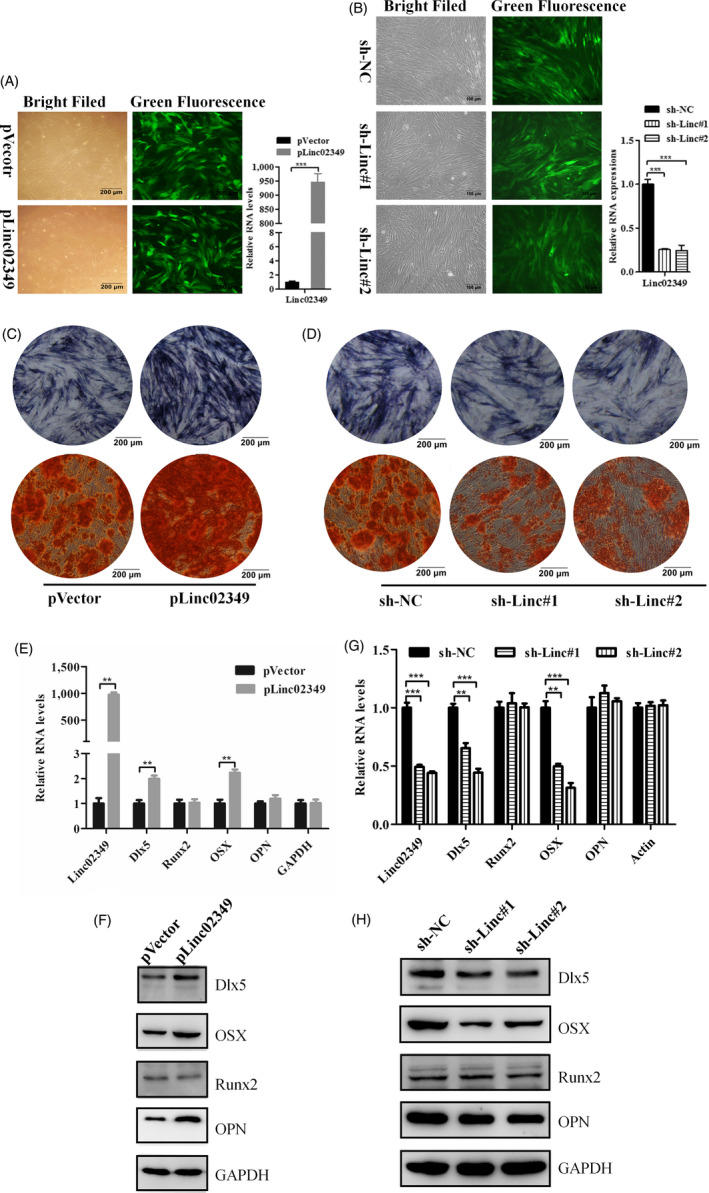
Linc02349 promotes osteogenesis of hUC‐MSCs. A, Left: Representative micrographs of GFP + hUC‐MSCs infected with linc02349 overexpression lentivirus (pLinc02349 group) and empty vector (pVector group) under ordinary and fluorescent light. Scale bars, 200 μm. Right: qPCR analysis confirming linc02349 overexpression efficiency. B, Left: micrographs of GFP + hUC‐MSCs infected with shlinc02349#1 lentivirus(sh‐linc#1group), shlinc02349#2 lentivirus (sh‐linc#2 group) and sh‐NC. Scale bars, 200 μm. Right: qPCR analysis confirming linc02349 knockdown efficiency. C, The alkaline phosphatase activities in pVector and plinc02349 groups were measured by ALP staining on day 7 of hUC‐MSCs osteogenesis (upper panel), and the calcified nodules were detected by alizarin red staining on day 21 (lower panel). Representative pictures showed that linc02349 overexpression promoted the osteogenesis. D, The alkaline phosphatase activities in sh‐NC, sh‐linc#1 and sh‐linc#2 groups were measured by ALP staining on day 7 (upper panel), and the calcified nodules were measured by alizarin red staining on day 21 (lower panel). Representative pictures showed that linc02349 knockdown inhibited the osteogenesis. E, qPCR showed that the mRNA levels of osteogenic marker genes Dlx5 and Osterix(OSX) were increased through overexpression of linc02349. F, Overexpression of linc02349 increased the protein level of Dlx5, OSX and OPN. G, Knockdown of linc02349 inhibited the mRNA level of Dlx5 and OSX. H, Knockdown of linc02349 decreased the Dlx5, OSX and OPN protein level during the osteogenesis. Values are mean ± SEM. n = 3. **P < .01; ***P < .001

To explore the function of linc02349 during osteogenesis, we used lentivirus‐mediated short hairpin RNAs to stably knockdown linc02349 and stably overexpress linc02349 in hUC‐MSCs and the established stably cell lines were verified with cell florescence intensity and qPCR analysis (Figure 2A,B). Notably, successful over expression of linc02349 accelerates the differentiation of hUC‐MSCs proved by the increased activity of alkaline phosphatase (ALP), an early marker of osteogenic differentiation, which was measured at day 7. Additionally, linc02349 overexpression remarkably increased calcium nodules, measured by alizarin red S staining assays at day 21 (Figure 2C). By contrast, knockdown of linc02349 results in significantly reduced the activity of ALP and decreased calcium nodule (Figure 2D).

The mRNA levels of several osteogenic marker genes were examined at day 7 of osteogenic differentiation, and Dlx5 and Osterix (OSX) were upregulated in linc02349‐overexpressed cells, whereas they were downregulated by linc02349 knockdown (Figure 2E). Moreover, Dlx5, OSX and OPN protein levels were increased in linc02349‐overexpressed cells (Figure 2F), while the low expression of them was observed in linc02349 knockdown cells at day 7 of osteogenic differentiation (Figure 2G,H). Collectively, these data suggested that linc02349 plays a positive role in osteogenic differentiation of hUC‐MSCs.

### 3.3 Linc02349 plays the role as a molecular sponge for miR‐25‐3p and miR‐33b‐5p

**Figure 3:**
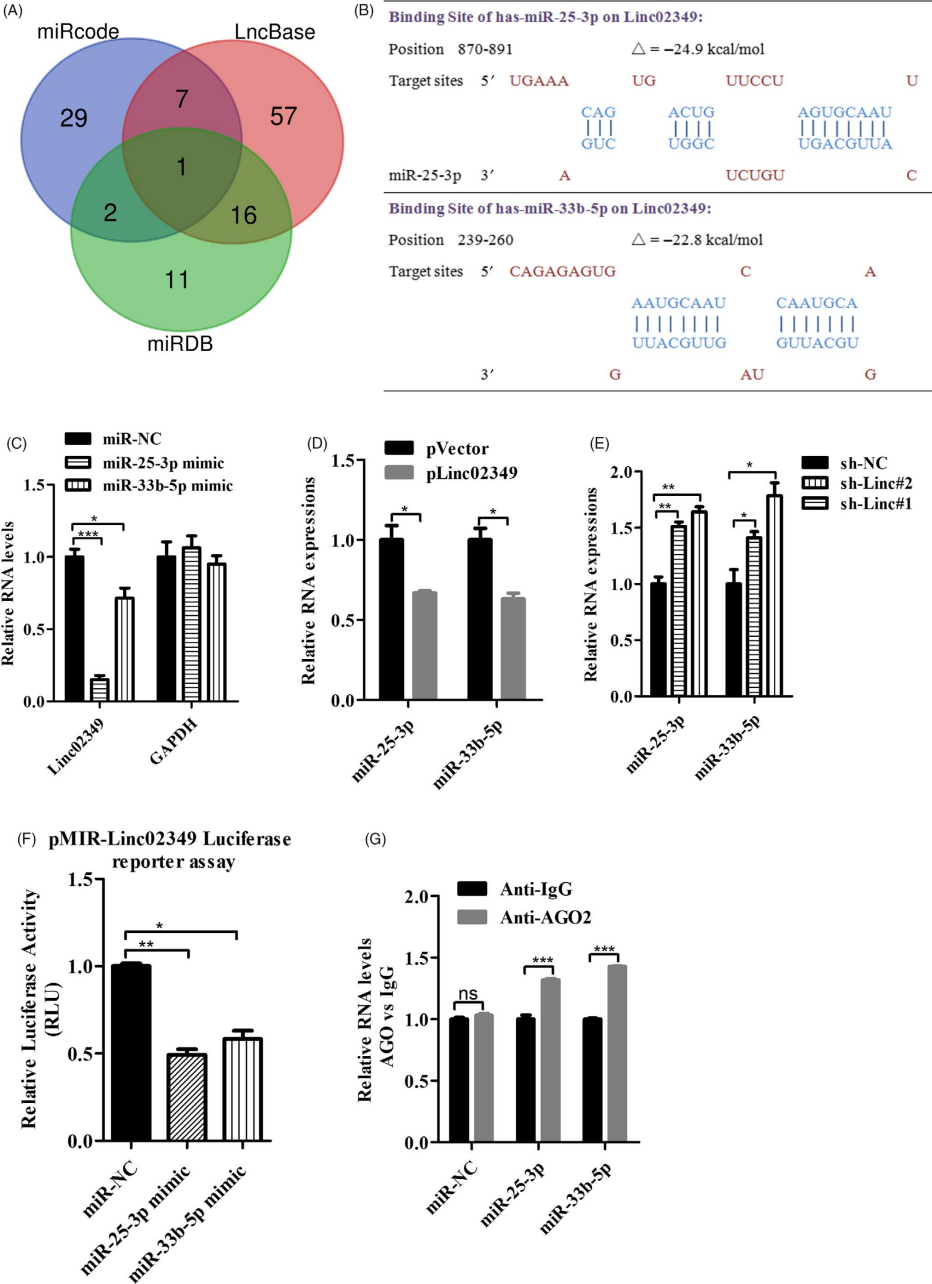
miR‐25‐3p and miR‐33b‐5p mediated the function of linc02349, respectively, in regulating the hUC‐MSCs osteogenic differentiation. A, Venn diagram showing the overlap of miRNAs who targets to linc02349 predicted by miRcode (light blue), LncBase (light red) and miRDB (light green). B, Schematic diagrams of the binding sites between miRNA and linc02349. C, The miR‐25‐3p and miR‐33b‐5p mimics significantly decreased the expression of linc02349 on day 7 of hUC‐MSCs osteogenesis. D, qPCR showed that miR‐25‐3p and miR‐33b‐5p were reduced during overexpression of linc02349. E, Knockdown of linc02349 increased the expression level of miR‐25‐3p and miR‐33b‐5p. F, miR‐NC, miR‐25‐3p or miR‐ 33b‐5p mimics was co‐transfected with pMIR‐lin02349 luciferase reporter into 293T cells, respectively, subsequently measured the luciferase fluorescence levels. The pRL‐TK vector was used as an internal control. The luciferase reporter assay showed that miR‐25‐3p and miR‐25‐3p reversely controlled the luciferase activity of pMIR‐linc02349. G, The association between linc02349, miRNA and Ago2 was ascertained by detecting cell lysates of miR‐NC, miR‐25‐3p or miR‐33b‐5p mimic transfected hUC‐MSCs using RIP‐qPCR with an Ago2 antibody. IgG was used as the control. The data are shown as mean ± SEM. N = 3. *P < .05; **P < .01; ***P < .001

Linc02349 is located slightly more in the cytoplasm than in the nucleus in differentiated cells (Figure 1E,F). Consistently, the neighbouring gens displayed no significant changes (Figure S3A). We hypothesized that linc02349 may functions as a ceRNA, resulting in the release of miRNAs targets. To assess that, three in silico bioinformatics softwares were used for predicting the miRNA candidates that targeting linc02349. We identified 39 miRNAs by miRCode, 30 miRNAs by miRDB and 81 miRNAs by LncBase (Figure 3A). Among them, miR‐25‐3p and miR‐33b‐5p were regarded as the most promising candidates on account of the literature datum and their lowest binding free energy (Figure 3B). miR‐25‐3p and miR‐33b‐5p mimics could suppress the linc02349 expression (Figure 3C). Interestingly, overexpression of linc02349 led to the decreased expression of miR‐33b‐5p and miR‐25‐3p (Figure 3D). In parallel, linc02349 knockdown in osteogenic induction cells elicited a significant increase of miR‐33b‐5p and miR‐25‐3p transcripts compared with NC group (Figure 3E). To probe that linc02349 was indeed targeted by them, a luciferase reporter vector harbouring linc02349 full‐length sequence (pMIR‐linc02349) was constructed and the luciferase assay was performed. The results showed that the group with miR‐25‐3p and miR‐33b‐5p mimics transfected significantly inhibited the luciferase activity of the constructed pMIR‐linc02349 vector compared with miR‐NC mimics‐transfected group (Figure 3F). To directly confirm that, we performed an Ago2 RIP‐qPCR analysis, which showed that linc02349 enrichment was higher in miR‐25‐3p and miR‐33b‐5p than that in the miR‐NC (Figure 3G). These data indicated that linc02349 directly “sponges” miR‐33b‐5p and miR‐25‐3p.

### 3.4 Linc02349 decoys miR‐25‐3p and miR‐33b‐5p to upregulate its target SMAD5 and Wnt10b, respectively

**Figure 4:**
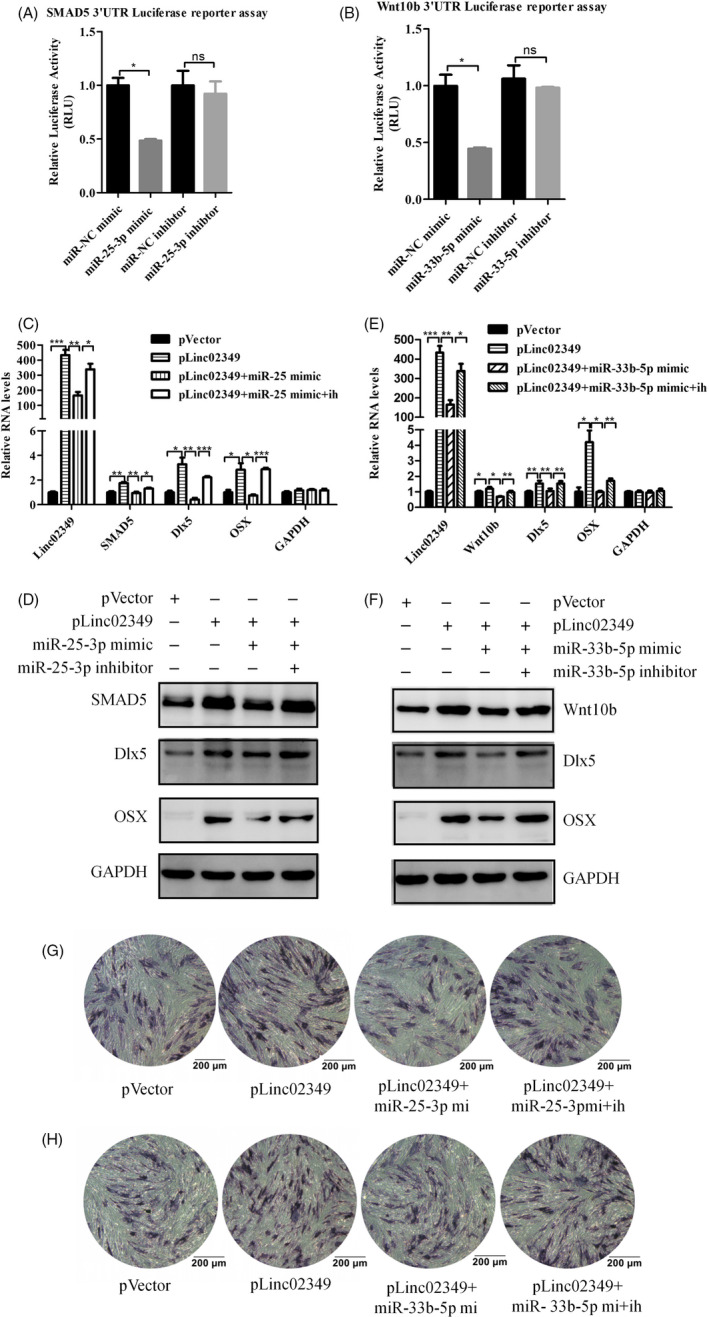
Linc02349 upregulated SMAD5 or Wnt10b by antagonized the functions of miR‐25‐3p or miR‐33b‐ 5p, respectively. A, miR‐25‐3p mimic negatively regulated the luciferase activity of SMAD5 3’UTR, and miR‐25‐3p inhibitor (ih for short) made no significant change in the luciferase activity. B, miR‐33b‐5p mimic negatively regulated the luciferase activity of Wnt10b 3’UTR, and miR‐33b‐5p inhibitor made no significant change in the luciferase activity. C, D, Overexpression of linc02349 increased the mRNA and protein levels of SMAD5, Dlx5 and OSX, which reversed by miR‐25‐3p mimic while rescued by miR‐25‐3p inhibitor. E, F, The mRNA and protein levels of SMAD5, Dlx5 and OSX increase by upregulation of linc02349 was reversed by miR‐33b‐5p mimics while rescued by miR‐33b‐5p inhibitor. G, H, The ALP activities were performed on day 3 of osteogenesis, which indicated that the alkaline phosphatase activity increase by upregulation of linc02349 was reversed by miR‐25‐3p or miR‐33b‐5p mimic (mi for short) while rescued by miR‐25‐3p or miR‐33b‐5p inhibitor (ih for short). The data are shown as mean ± SEM. n = 3. *P < .05; **P < .01; ***P < .001

To clarify the potential mechanisms of miR‐25‐3p and miR‐33b‐5p with linc02349, we explored the candidate downstream targets based on bioinformatics prediction (TargetScan and StarBase). The binding sequence of miR‐25‐3p was recognized and predicted in 3’UTR of SMAD5 mRNA, and miR‐33b‐5p predicted targets to the 3’UTR of Wnt10b mRNA (Figure S4A). SMAD5 is a key regulator of BMP/SMAD signalling and promotes osteogenic differentiation in mesenchymal stem cells, and Wnt10b promotes the expression of osteogenic marker genes, such as Dlx5 and OSX.^25^, ^26^ We hypothesized that linc02349 might competitively absorb miR‐25‐3p and miR‐33b‐5p to release its target SMAD5 and Wnt10b, respectively. We firstly generated a luciferase reporter harbouring SMAD5 3’UTR or Wnt10b 3’UTR sequence and applied for luciferase reporter assay. Luciferase reporter assays showed that overexpression of miR‐25‐3p repressed luciferase activity in hUC‐MSCs transfected with the SMAD5 3'‐UTR reporter vector, while no obvious inhibition was observed in cells co‐transfected with miR‐25‐3p inhibitor and SMAD5 3'‐UTR reporter plasmid (Figure 4A). The luciferase activity of Wnt10b 3’UTR reporter vector was decreased upon miR‐33b‐5p mimics transfection and was rescued by miR‐33b‐5p inhibitors (Figure 4B).

Moreover, transfection of miR‐25‐3p or miR‐33b‐5p mimics abolished linc02349‐mediated elevated SMAD5 and Wnt10b mRNA and protein expression and blocked downstream targets, including Dlx5 and OSX, while miR‐25‐3p or miR‐33b‐5p inhibitors were sufficient to restore the change (Figure 4C‐F). The ALP staining experiments were examined at day 3 of osteogenic differentiation showed that the activity of alkaline phosphates was obviously increased by overexpression of linc02349, but which promotional effects could offset by miR‐25‐3p/33b‐5p overexpression, and miR‐25‐3p or miR‐33b‐5p inhibitors could largely rescue this effect (Figure 4G,H). Collectively, these data confirmed that linc02349 functions as a molecular sponge for miR‐25‐3p and miR‐33b‐5p to facilitate expression of SMAD5 and Wnt10b.

### 3.5 Overexpression of linc02349 enhanced hUC‐MSCs differentiation in vivo

**Figure 5:**
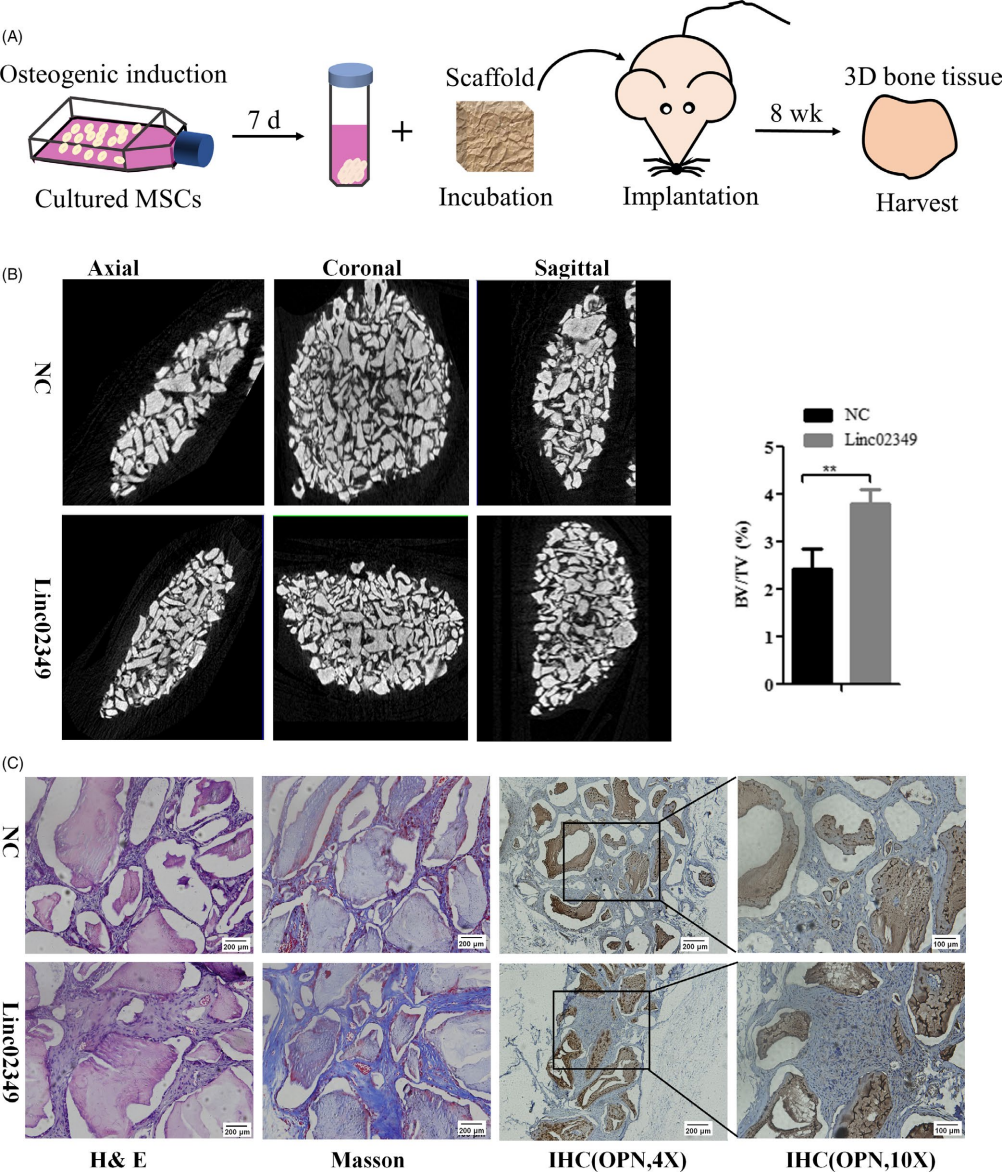
Overexpression of linc02349 enhanced hUC‐MSCs differentiation in vivo. A, Schematic diagram illustrating the experiment process. B, Left: Representative three dimensions micro‐CT images of bone formation in Bio‐Oss Collagen scaffolds of linc02349 (n = 3) and NC groups (n = 3). Right: Quantitative analysis of BV/TV (bone volume/tissue volume, %). The data were expressed as mean ± SD. **P < .01. C, H&E staining, Masson's trichrome staining and immunohistochemical staining of OPN in NC, linc02349 groups

We next sought to determine the function of linc02349 during osteogenesis in vivo. HUC‐MSCs were infected with linc02349 overexpression or NC lentivirus and cultured in osteogenic‐induced medium for 7 days in vitro. Then the cells were seeded on Bio‐Oss Collagen Scaffolds and the scaffolds were implanted into the subcutaneous space of nude mice (five mice per group). Eight weeks later, the implantation samples were harvested and analysed (Figure 5A). The representative three dimensional micro‐CT images indicated that linc02349 overexpression group had a greater density of bone form. Micro‐CT showed the BV/TV was significantly increased in the linc02349 over expressing group (Figure 5B). Compared with NC group, the amount of bone tissue and bone trabeculae in H&E staining and collagen organization with blue colour in Masson's trichrome staining was significantly higher. In addition, immunohistochemical staining (IHC) analysis for OPN revealed that both the range and intensity of the stained granules were increased in the linc02349 overexpression group than control one (Figure 5C). Collectively, the results suggest that linc02349 contributes to the osteogenesis of hUC‐MSCs in vivo.

### 3.6 Linc02349 is modulated by STAT3 in the process of differentiated hUC‐MSCs to osteoblasts

**Figure 6:**
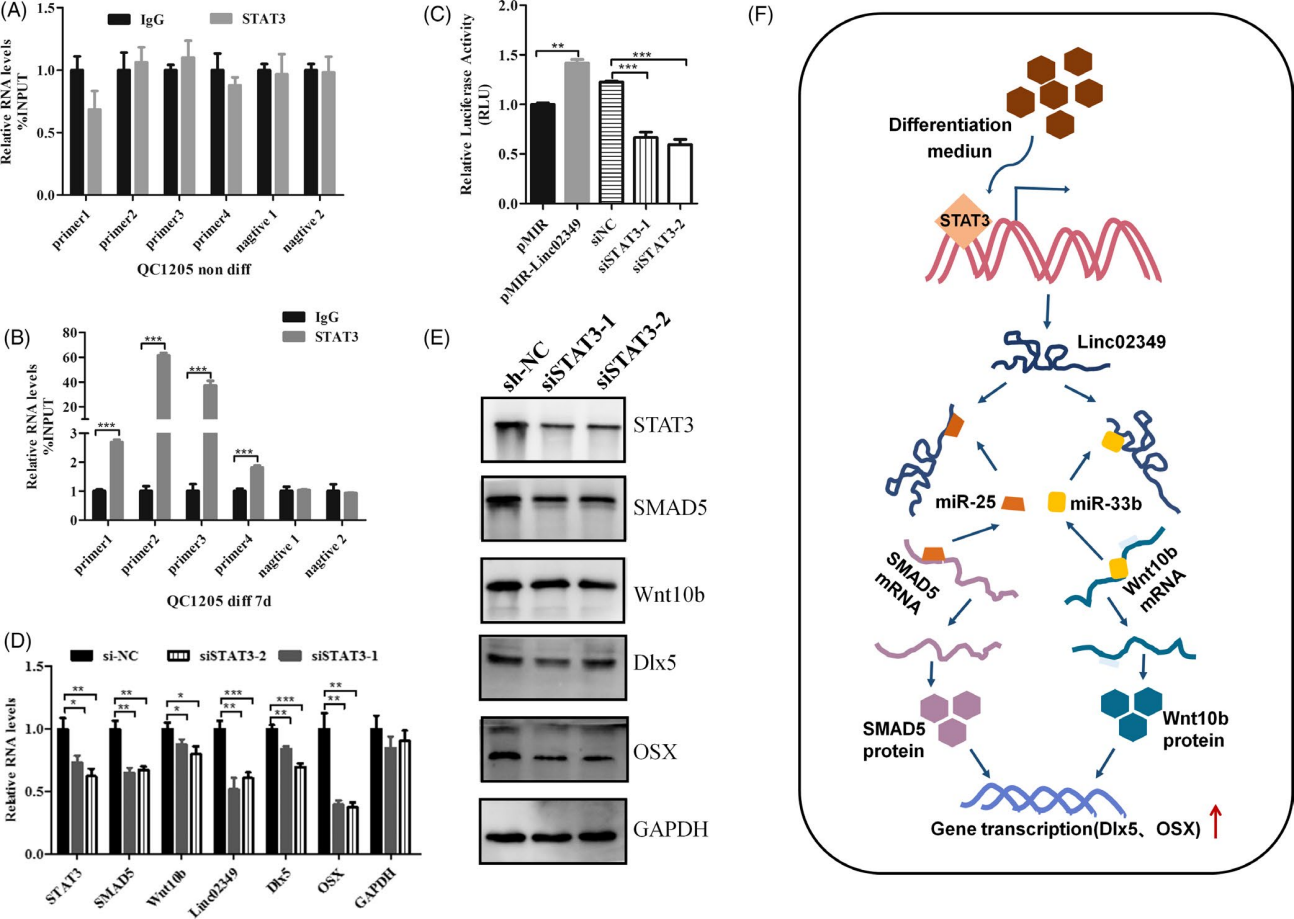
STAT3 promotes linc02349 transcriptional activities. A, STAT3 binding to the linc02349 promoter region was assessed by ChIP‐qPCR on day 0 of osteogenesis. B, ChIP‐qPCR assay of STAT3 in the linc02349 promoter region on day 7 of osteogenesis. C, Luciferase assay was conducted to examine the activities of linc02349 promoter on day 7 of osteogenesis following the pMIR‐REPORTTM vector transfected only, or pMIR‐linc02349‐promoter luciferase reporter co‐transfected with si‐NC, si‐STAT3‐1 or si‐STAT3‐2. The pRL‐TK vector is used as an internal control reporter vector. D, The qPCR was performed at 24 h after transfected with si‐NC, si‐STAT3‐1 or si‐STAT3‐2. Data were normalized to Actin. The data were expressed as mean ± SEM. n = 3. *P < .05; **P < .01; ***P < .001. E, The Western blotting was performed at 48 h following transfected with si‐NC, si‐STAT3‐1 or si‐STAT3‐2. F, Graphical abstract of how linc02349 regulates hUC‐MSCs osteogenesis. Linc02349 promotes the osteogenic differentiation of hUC‐MSCs by competitively binding miR‐25‐3p and miR‐33b‐5p, leading to the increased expression of SMAD5, Wnt10b and activation of OSX and Dlx5 signalling. Meanwhile, activated STAT3 promotes the transcriptional of linc02349

We then explored the underlying mechanism for linc02349 upregulation in osteogenic differentiation of hUC‐MSCs. Bioinformatics analysis^27^ of the promoter sites (−2000 to 200 bp) of linc02349 predicted two DNA‐binding elements for c‐JUN and STAT3 (Figure S5A,B). We established recombinant plasmid including the promoter region of linc02349 and designed eight pairs of primers for the linc02349 promoter region, then conducted ChIP assay. Interestingly, we found that STAT3 are enriched in the promoter region of linc02349 during osteogenesis, whereas c‐JUN showed no enrichment (Figure 6A,B, Figure S5C). We then validated that STAT3 silencing remarkably reduced the relative luciferase activity of the promoter (Figure 6C). Consistently, STAT3 knockdown decreased linc02349, SMAD5, Wnt10b, OSX and Dlx5 expression at mRNA and protein levels (Figure 6D,E). Collectively, these data suggested that activated STAT3, at least, in part, resulted in the upregulation of linc02349 in the process of osteogenic differentiation.

## 4 DISCUSSION

Osteoporosis has been listed as one of the “three killers” that are harmful to the health of the elderly by the World Health Organization together with diabetes and cardiovascular disease. At present, most of the commonly used drugs in clinic are bone absorption inhibitors. However, drug treatment of osteoporosis is not ideal. Although it can delay the progress of osteoporosis, it cannot make up for the lost bone mass.^28^ Using stem cell self‐renewal and differentiation ability to treat osteoporosis may be the most potential way of bone repair, which is expected to solve the medical problems of bone regeneration and repair.^29^ In recent years, biological scaffolds have made rapid progress.^30^, ^31^, ^32^, ^33^ It is particularly urgent to clarify the mechanism of stem cell differentiation and develop new strategies to induce stem cell differentiation. Recent advances have demonstrated that inducing mesenchymal stem cells differentiation into osteoblasts and promoting bone formation might be a new therapeutic approach.^34^, ^35^ Currently, hUC‐MSCs have been identified as promising sources of mesenchymal stem cells according to their capacity of self‐renewal, multipotency and low immunogenic properties^36^ and better proliferative ability than other mesenchymal stem cells.^37^, ^38^, ^39^ Notably, some groups have validated the application of hUC‐MSCs in cartilage degradation by promoting chondrogenesis and inhibiting IL‐1β‐induced inflammatory response.^40^ In this study, we defined a lncRNA termed linc02349 that promotes the mesenchymal stem cell differentiation to osteoblasts acts a as molecular sponge for miR‐25‐3p and miR‐33b‐5p to modulate SMAD5 and Wnt10b expression and trigger Dlx5/OSX signalling to initiate osteogenesis of hUC‐MSCs. This study will provide experimental basis for hUC‐MSCs to be applied in the therapy of bone degradation.

Runx2 is the most upstream transcription factor essential for osteoblast differentiation. It regulates the expression of OSX, the protein of which is a crucial transcription factor for osteoblast differentiation. Dlx5 is the upstream of Runx2/OSX and plays positive role by Runx2‐dependent or Runx2‐independent manner during osteogenic differentiation. The enhancer of Runx2 is activated by an enhanceosome composed of Dlx5/6, Mef2, Tcf7, Ctnnb1, Sox5/6, Smad1 and OSX.^41^ In another aspect, the BMP‐2‐induced OSX expression is mediated by Dlx5 but is independent of Runx2.^42^ In our study, there was no significant difference for Runx2 level with linc02349‐mediated upregulation of Dlx5 and OSX even though we repeated these experiments. In this case, these results supported the conclusion that Dlx5 regulates OSX independent of Runx2.

MicroRNAs regulate gene expressions via functional RNA‐induced silencing complex by targeting mRNAs.^43^ And the lncRNA with shared miRNA‐binding sites could compete for post‐transcriptional control.^44^ In this study, we demonstrate that miR‐25‐3p targets to SMAD5, miR‐33b‐5p targets to Wnt10b, thereby inhibiting their gene expression. Yet linc02349 abolished the miR‐25‐3p/33b‐5p‐mediated biological effects. Together, we revealed the role of linc02349 as a ceRNA for miR‐25‐3p/33b‐5p. Wnt10b shifts cell fate towards the osteoblast lineage by induction of the osteoblastogenic transcription factors SMAD5, Dlx5 and OSX.^25^, ^45^, ^46^ SMAD5 is the gene involving BMP signal and promotes osteogenic differentiation of MSC.^26^ Together, we revealed the role of linc02349 as a ceRNA for miR‐25‐3p/33b‐5p.

In this study, the upstream transcript factor STAT3 was identified to regulate the expression of linc02349. It was found that STAT3 are enriched in the promoter region of linc02349 during osteogenesis by ChIP assay. The luciferase assay also demonstrated that STAT3 activated the expression of linc02349. This elucidated that STAT3 is one of the upstream regulator. STAT3 has been reported to play important role during the differentiation of multiple and pluripotent stem cells.^47^ STAT3 induces muscle stem cell differentiation by interaction with myoD.^48^ STAT3 induces mouse embryonic stem cell differentiation into cardiomyocytes.^49^ Glucocorticoid receptor can be tethered to the DNA via its interaction with a STAT3.^50^ Activated STAT3 was associated with ligand‐bound glucocorticoid receptor to form a transactivating signalling complex and acted as a transcriptional co‐activator in this signalling.^51^ Therefore, ligand‐bound glucocorticoid receptor may recruit STAT3 to the promoter region and activate the transcription of linc02349. This is consistent with the results that it is dexamethasone, a kind of glucocorticoid, plays a key role in inducing the expression of linc02349.

In summary, we revealed that linc02349 promoted hUC‐MSCs osteogenesis by acting as a ceRNA for miR‐25‐3p resulting in upregulation of SMAD5 and for miR‐33b‐5p resulting in the increasing of Wnt10b expression, which further activates the Dlx5 and OSX, enhancing the osteogenic differentiation of hUC‐MSCs. In addition, it was also revealed that linc02349 is regulated by upstream transcription factor STAT3 activated by the induction of glucocorticoid receptor signalling (Figure 6F). The present study may illustrate the function of the lncRNA‐microRNA‐mRNA ceRNA network in MSCs differentiation, and linc02349 may be a potentially useful for osteoporosis diagnosis and therapies.

## CONFLICT OF INTERESTS

The authors declare that there are no competing interests.

## AUTHOR CONTRIBUTION

Lihua Cao and Wei Liu conducted most of the experiments. Zi Zou, Dan Gao, Tiantian He, Ying Liu and Haotian Ma conducted the statistical analysis and animal experiments. Lihua Cao, Yancheng Zhong, Yanru Zhang and Yuqing Mo wrote, reviewed and edited the manuscript. Shuping Peng and Cijun Shuai contributed the project design, administration and financial support.

## Supporting information

FigS1

FigS2

FigS3

FigS4

FigS5

Table S1

## Data Availability

All data generated or analysed during this study are included in this article, which are available upon request by contact with the corresponding author.
