# Supplementary figures and images for: Linc02349 promotes osteogenesis of human umbilical cord‐derived stem cells by acting as a competing endogenous RNA for miR‐25‐3p and miR‐33b‐5p
Source: Cell Prolif. 2020 Apr 29;53(5):e12814. doi: 10.1111/cpr.12814 (PMC7260076; doi:10.1111/cpr.12814)

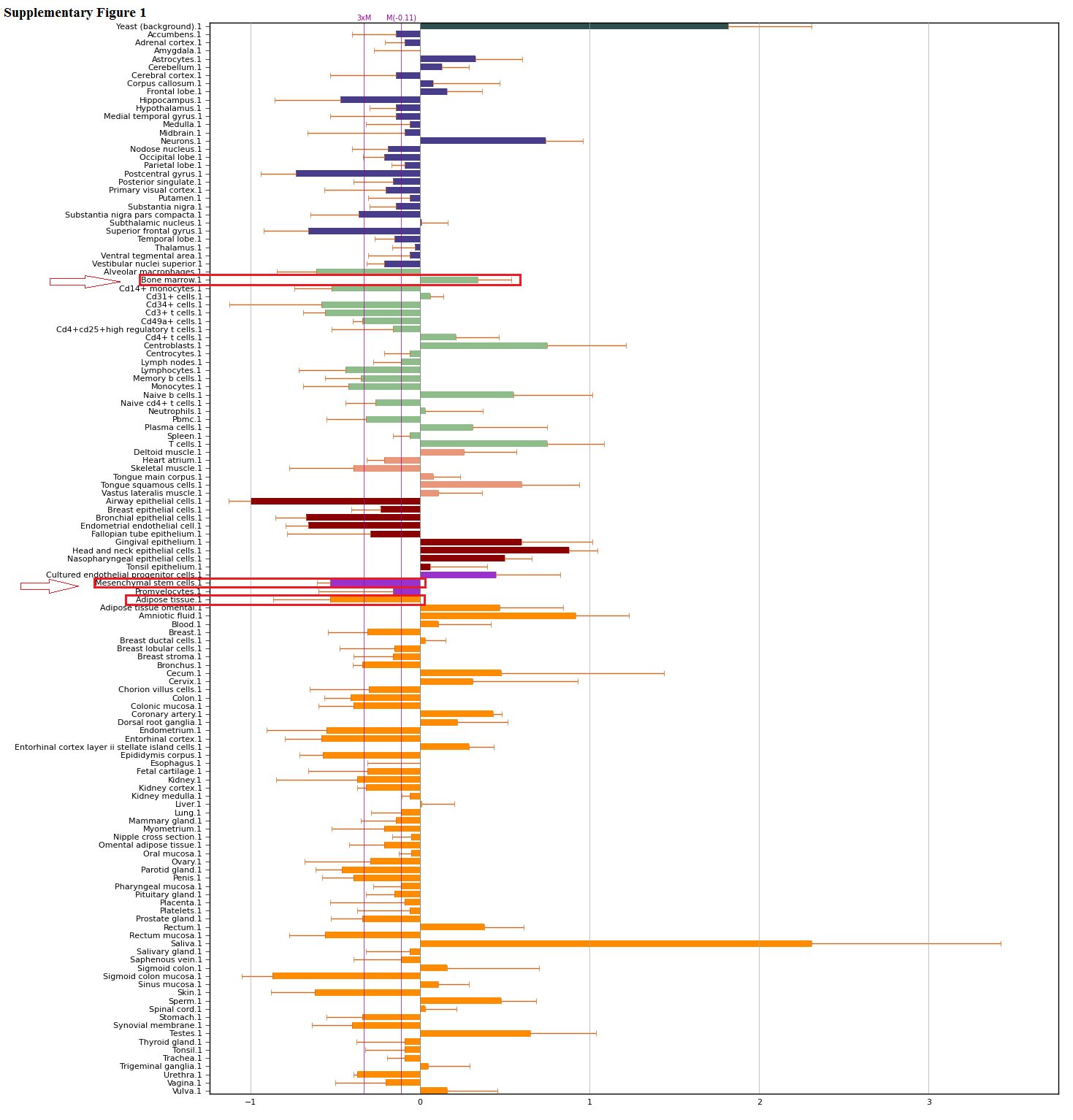

Supplement: Supplementary file 1 — FigS1 [file CPR-53-e12814-s001.tif]

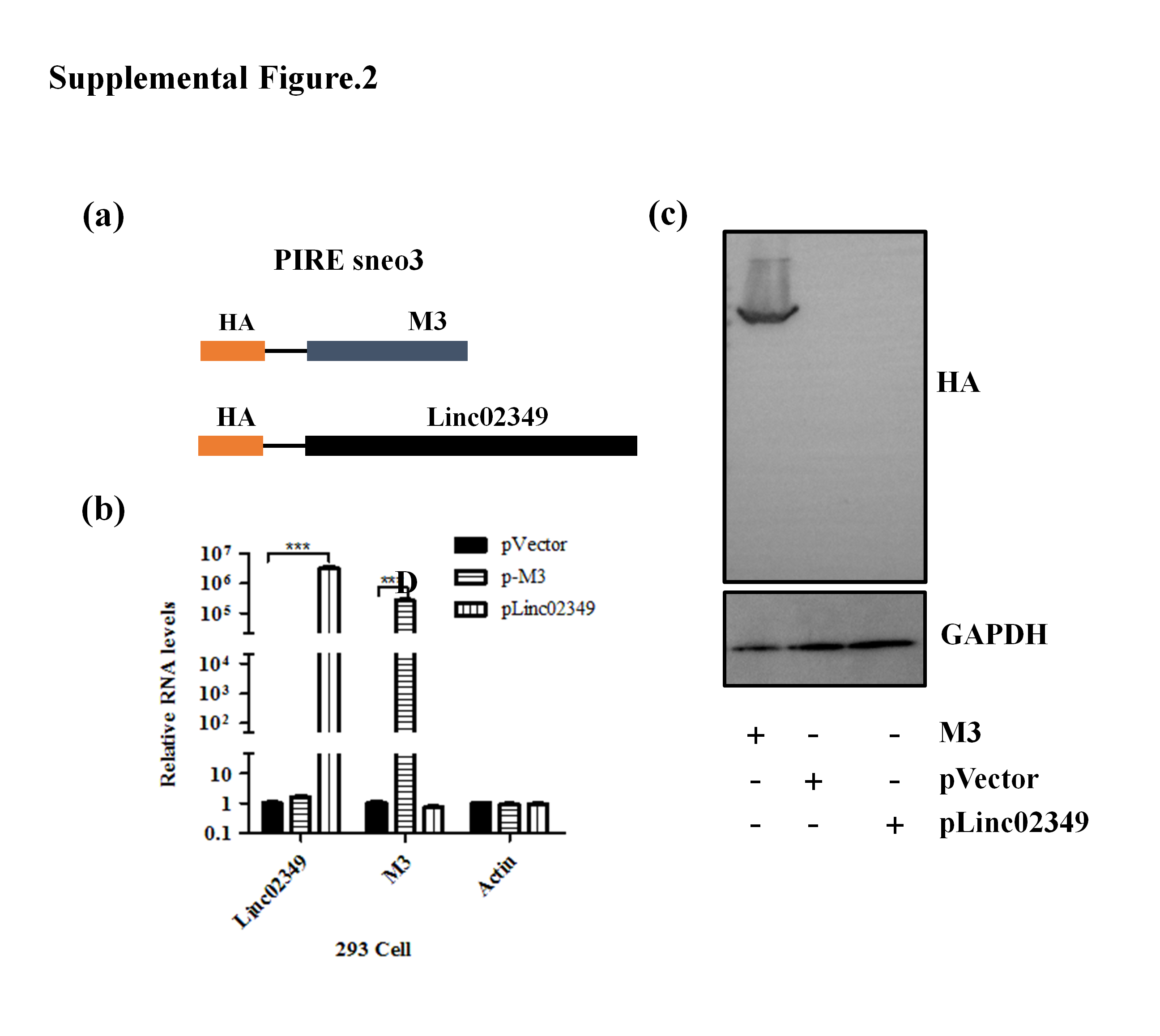

Supplement: Supplementary file 2 — FigS2 [file CPR-53-e12814-s002.tif]

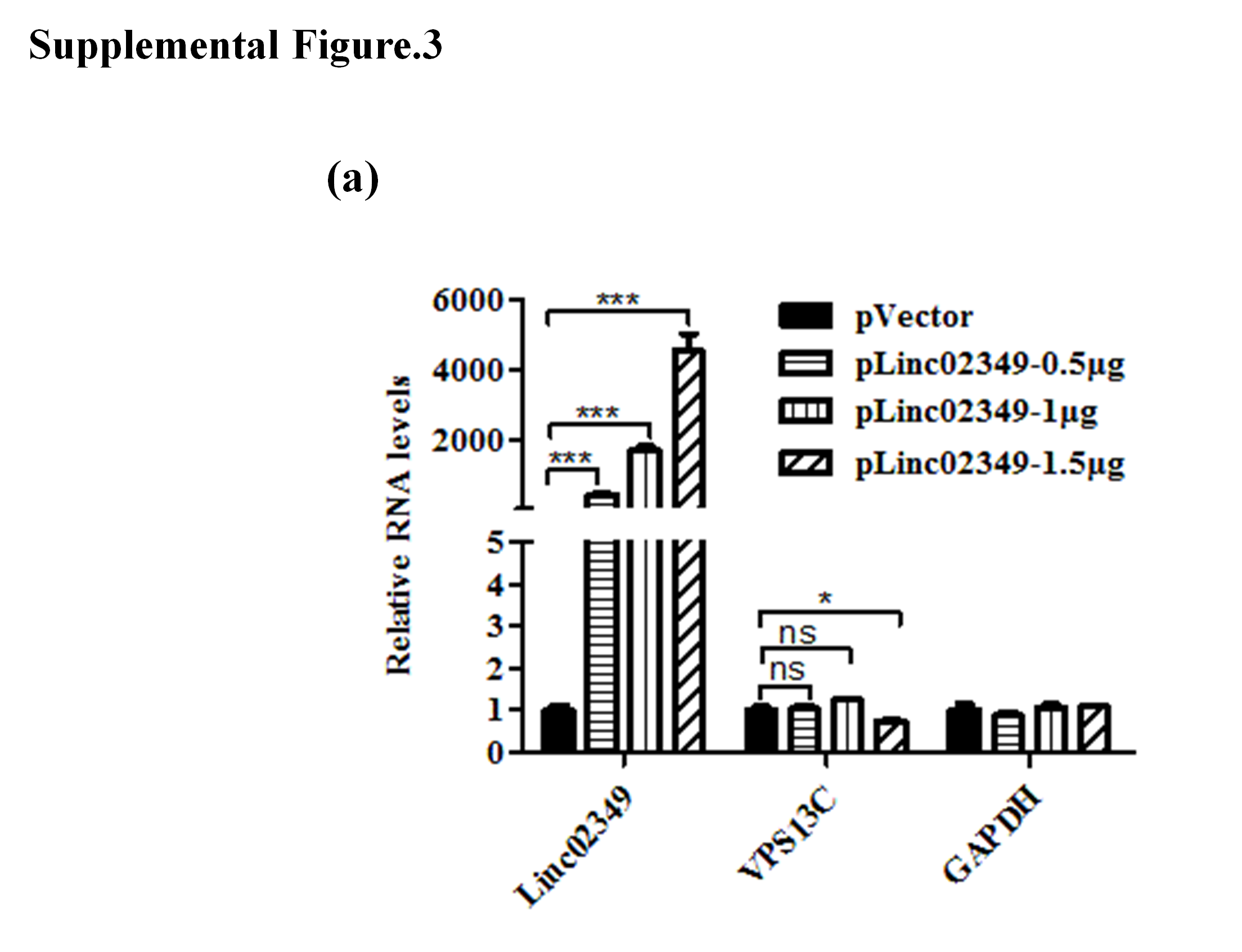

Supplement: Supplementary file 3 — FigS3 [file CPR-53-e12814-s003.tif]

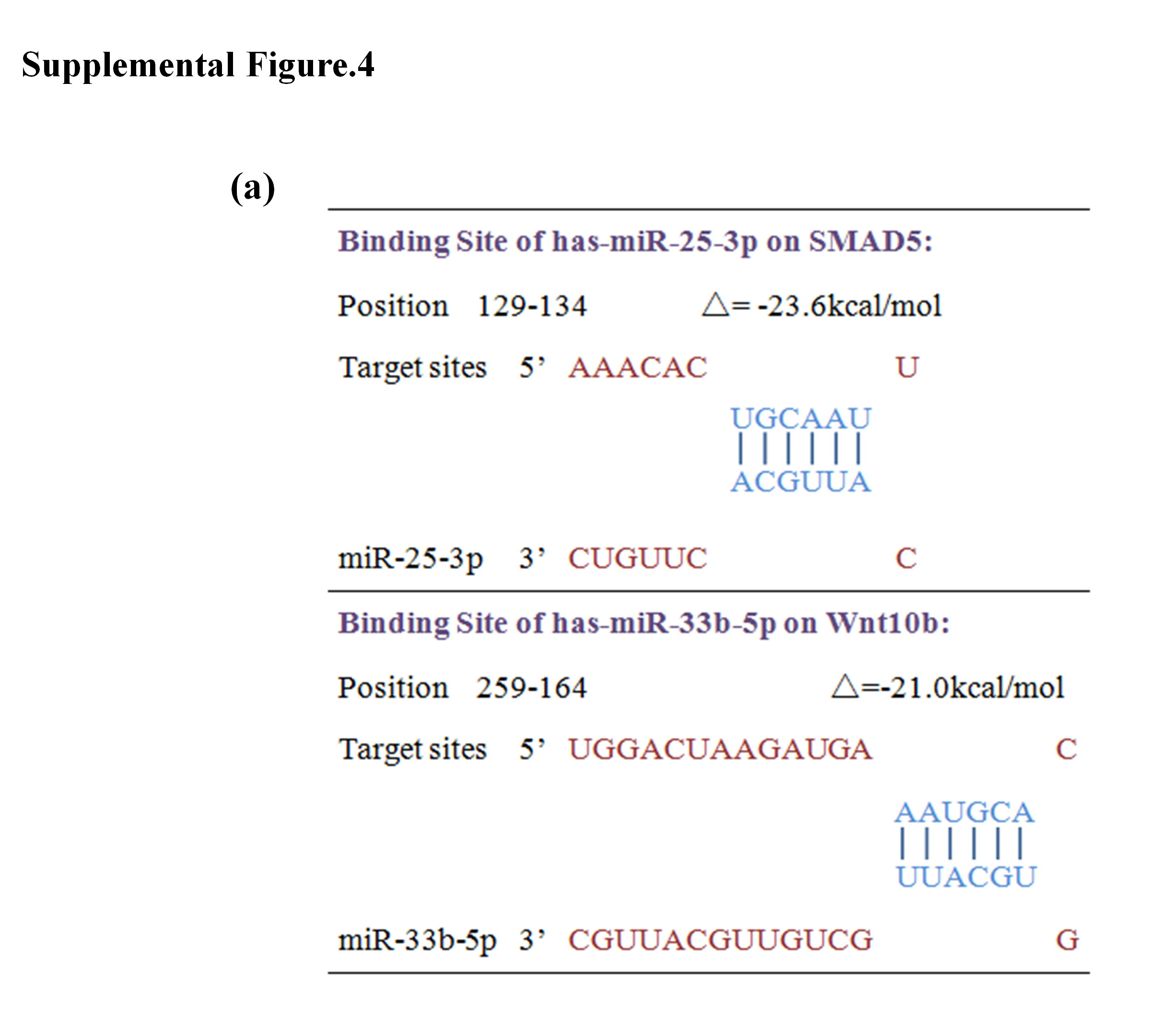

Supplement: Supplementary file 4 — FigS4 [file CPR-53-e12814-s004.tif]

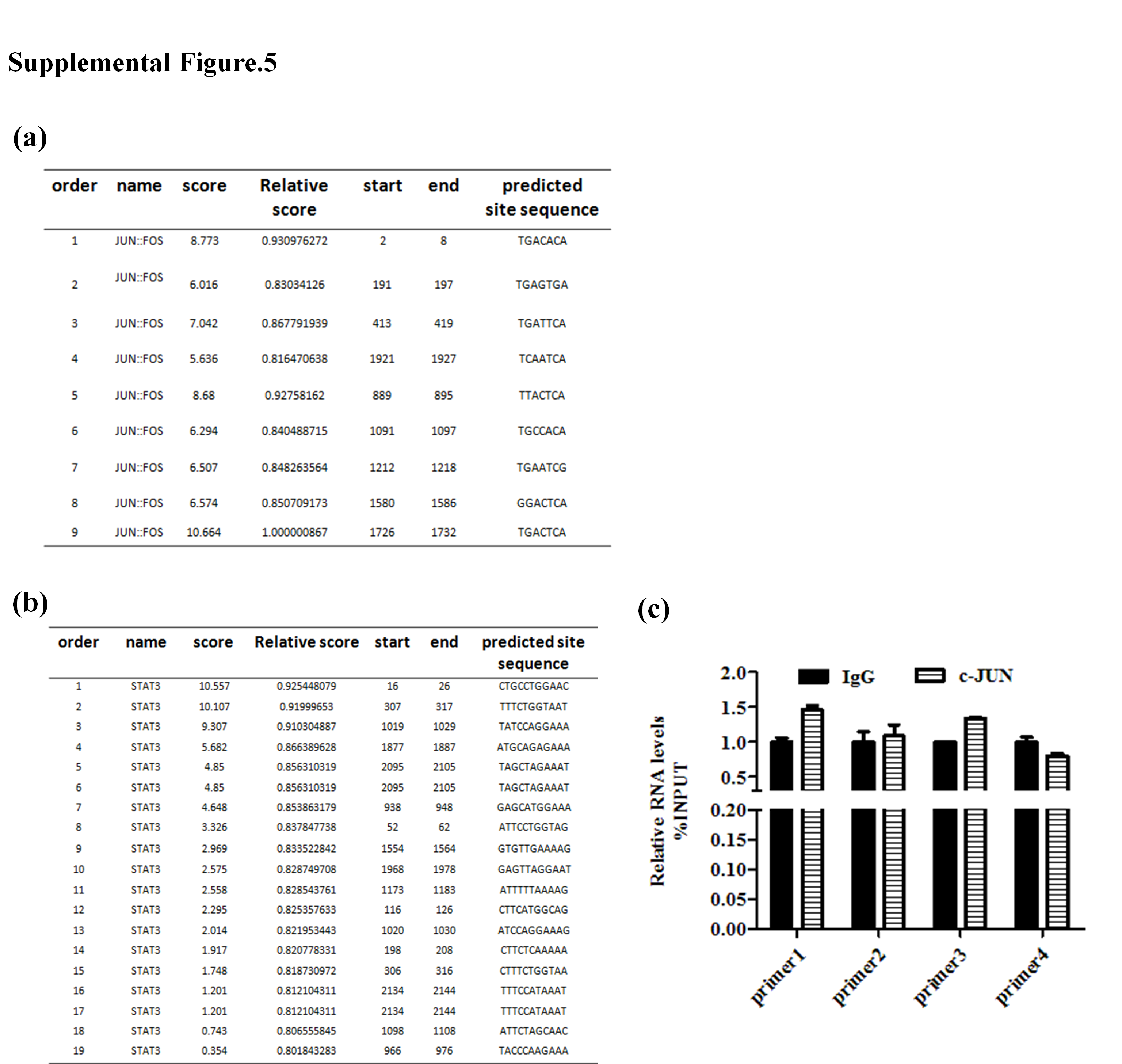

Supplement: Supplementary file 5 — FigS5 [file CPR-53-e12814-s005.tif]
