# Supplementary material for: Linc02349 promotes osteogenesis of human umbilical cord‐derived stem cells by acting as a competing endogenous RNA for miR‐25‐3p and miR‐33b‐5p
Source: Cell Prolif. 2020 Apr 29;53(5):e12814. doi: 10.1111/cpr.12814 (PMC7260076; doi:10.1111/cpr.12814)
Supplement: Supplementary file 6 — Table S1 [file CPR-53-e12814-s006.docx]

**Supplementary Table S1: Primers for vector construction, qPCR, ChIP-qPCR and siRNAs**

| **Name** | **Forward Primer (5'-3')** | **Reverse Primer (5'-3')** |
| --- | --- | --- |
| **Primers for HA-Linc02349 construct** | | |
| HA-Linc02349 | ATAGCCTCGCGGATCCTCTGGTTTCTATATTAATTT | CAGGATATCATGTACCCATACGATGTTCCAGATTACGCTCTTTCATAATTTGTTCTAGG |
| **Primers for shLinc02349 construct** | | |
| shLinc02349#1  shLinc02349#2 | GATCTCCTTGGTGCAGCTGAGTTTTTCAAGAGAAAACTCAGCTGCACCAAGGTTTTTA  GATCTGCTCTTTCAGGAAGATTTATTCAAGAGATAAATCTTCCTGAAAGAGCTTTTTA | AGCTTAAAAACCTTGGTGCAGCTGAGTTTTCTCTTGAAAAACTCAGCTGCACCAAGGA  AGCTTAAAAAGCTCTTTCAGGAAGATTTATCTCTTGAATAAATCTTCCTGAAAGAGCA |
| **Primers for luciferase reporter gene vector construct** | | |
| pMIR-Linc02349 | CCGACTAGTCATTCCTGGTAGTTTTGATGAAGATGG | TTACGCGTCTACCAACCAATGCAAACTG |
| pMIR-Wnt10b 3’UTR | TTAACGCGTCCCTTCCCACCCTTCCTT | CCGACTAGTAGGGTCAGCCTTACCTTGGG |
| pMIR-SMAD5 3’UTR | CATGCCGGCGACTTGGACTTAGATGCTTATCC | CATACGCGTCCATCCACTGGCTGCACATTG |
| **Primers for overexpresson of Linc02349 construct** | | |
| pCDH-linc02349 | CCGGAATTCCTTTCATAATTTGTTCTAGGGC | GCGCGCGGATCCTCTGGTTTCTATATTAATTTAT |
| **Primers for qPCR** | | |
| Linc02349 | ATGCAATGTTTCGATCATGG | ACCATCCCCAGGGTCTTAGT |
| Dlx5 | ACCATCCGTCTCAGGAATCG | ACCTTCTCTGTAATGCGGCC |
| Wnt10b | CGGTTGTGGGTATCAATGAAGA | CATCCAGGCACGAATGCGA |
| SMAD5 | CCAGCAGTAAAGCGATTGTTGG | GGGGTAAGCCTTTTCTGTGAG |
| STAT3 | ATCACGCCTTCTACAGACTGC | CATCCTGGAGATTCTCTACCACT |
| VPS13C | TGTGGAAAAATTGGCAACTCAAG | CCCAGTGTGACACCAAATGAA |
| OPN | CATCACCTGTGCCATACCAG | GTCATGGCTTTCGTTGGACT |
| Runx2 | GAGTGGACGAGGCAAGAGTT | GAGGCGGTCAGAGAACAAAC |
| OSX | CTTGTGCCTGATACCTGCACT | TCACTCTACCTGACCCGTCATC |
| Actin | TCACCAACTGGGACGACATG | GTCACCGGAGTCCATCCGAT |
| GAPDH | AACGGATTTGGTCGTATTGG | TTGATTTTGGAGGGATCTCG |
| U6 | CTCGCTTCGGCAGCACA | AACGCTTCACGAATTTGCGT |
| **Primers for RIP** | | |
| Linc02349 | ATGCAATGTTTCGATCATGG | ACCATCCCCAGGGTCTTAGT |
| GAPDH | AACGGATTTGGTCGTATTGG | TTGATTTTGGAGGGATCTCG |
| **Primers for ChIP** | | |
| STAT3 P1 | CATTCCTGGTAGTTTTGATGAAGATGG | CAAGGATTGAGCTTTTTGAG |
| STAT3 P2 | GTAAAACATGGAGGACAGAGG | ACCCAACATGAAGATCTGAAACC |
| STAT3 P3 | CCTGCTCCTCAATTTCCCCATG | CAACTGAGTGTGTGGCGTTC |
| STAT3 P4 | GGGAGCAACTGAAAACAAAATC | TACACGATTCACTCCTTTAGG |
| c-JUN P1 | GCTCTCTGAACAACACAATCATCA | GCACTAAACTTCCTTAGTTCC |
| c-JUN P2 | GGTTGGTTTCAGATCTTCATGTTGG | TTAGGGGCTATGGCAAGATGT |
| c-JUN P3 | CTGTCCCTCTGCAAGCCCAAGAGGTAA | GGGTAGTATAGCTTCATCTCTC |
| c-JUN P4 | TTAGCGGAACTTCATGCCACAA | CAGATGAGAAAAATTGGTTTTGC |
| Negative P1 | CCACGTTGCCTCCAGAAGAG | CAGAGTGGTGCTTAGAGGAA |
| Negative P2 | CTCTCCCCTCTGAATGCCTA | TGCAGCTGGTGCCACCCTTTGA |
| GAPDH | CGGGATTGTCTGCCCTAAT | GCACGGAAGGTCACGATGT |

Abbreviations: Runx2, Runt related transcription factor 2; OSX, Osterix/SP7; OPN,Osteopontin; Dlx5, Distal-less homeobox 5.
