# Supplementary material for: Linc02349 promotes osteogenesis of human umbilical cord‐derived stem cells by acting as a competing endogenous RNA for miR‐25‐3p and miR‐33b‐5p
Source: Cell Prolif. 2020 Apr 29;53(5):e12814. doi: 10.1111/cpr.12814 (PMC7260076; doi:10.1111/cpr.12814)
Supplement: Supplementary file 7 [file CPR-53-e12814-s007.docx]

**Supplementary figure legends**

**Supplemental Fig.1** Related to Figure 1. The expression of linc02349 gene in diverse normal human tissues from BioGPS. The expression of linc02349 in bone marrow, adipose tissue, and mesenchyma stem cells are labeled with red rectangle.

**Supplemental Fig.2** Linc02349 has no coding potentiality. Associated with Figure 1. **(a):**Full length linc02349 was cloned into the vector PIRE sneo3. M3 served as a positive control. An anti-HA antibody was for probe the transcribed proteins. **(b):** Relative RNA levels of linc02349 and M3 detected by qPCR in HEK293 cells after tranfected with empty vector, plinc02349, or pM3 plasmid respectively. **(c):** Western blotting analysis was performed to detect the transcribed proteins.

**Supplemental Fig.3** Related to Fig.3. The expression levels of linc02349 and VPS13C were examined by qPCR assay at 24h. Data were normalized to β-Actin.

**Supplemental Fig.4** **(a):** Schematic diagrams of the binding sites between SMAD5/Wnt10b and linc02349.

**Supplemental Fig.5** The binding sites between transcription factors and linc02349. Related to Fig.6. **(a and b):** The STAT3 and c-JUN are shown strong indications binding to linc02349 promoter region. The data were derived from the JASPAR database(<http://jaspar.genereg.net/>)**. (c):** c-JUN binding to the linc02349 promoter region was assessed by ChIP-qPCR on day 7 of osteogenesis.
